# Supplementary material for: Hippocampal and Parahippocampal Gray Matter Structural Integrity Assessed by Multimodal Imaging Is Associated with Episodic Memory in Old Age
Source: Cereb Cortex. 2020 Nov 5;31(3):1464–77. doi: 10.1093/cercor/bhaa287 (PMC7869080; doi:10.1093/cercor/bhaa287)
Supplement: Koehncke_et_al_Grey_Matter_Integrity_Supplement_R1_200907_bhaa287 [file koehncke_et_al_grey_matter_integrity_supplement_r1_200907_bhaa287.pdf]

Supplementary materials to article

Hippocampal and Parahippocampal Grey Matter Structural Integrity Assessed by Multimodal Imaging Is Associated with Episodic Memory in Old Age

Ylva Köhncke, Sandra Düzel, Myriam C. Sander, Ulman Lindenberger, Simone Kühn[+] &

Andreas M. Brandmaier[+]

[+]Joint last authorship

Table of Contents

1. **Episodic memory task descriptions** ... 2
Verbal Learning and Memory test (VLMT) ... 2
Face-Profession task ... 2
Scene Encoding ... 3
Object-Location task ... 3
2. **Fit indices and parameter estimates of final models** ... 4
Table S1. Fit indices and parameter estimates for the correlation model ... 4
Table S2. Fit indices and parameter estimates for the correlation model with age, education, and sex as covariates ... 7
Table S3. Fit indices and parameter estimates for the regression model ... 10
3. **Sex differences in variables** ... 13
Table S4: Sex differences in all variables of interest ... 13
4. **Sex differences in association structures** ... 14
Figure S1: First-order correlations among all variables by sex ... 14
Table S5: Measurement invariance tests for the brain-only MTMM-model ... 16
Table S6: Measurement invariance tests for the episodic memory measurement model ... 16
Table S7: Associations between episodic memory and each of the ROI-wise integrity factors by sex. from a multigroup model ... 17
Table S8: Associations between episodic memory and each of the ROI-wise integrity factors by sex. from a multigroup model with age and education as covariates ... 17
5. **Measurement invariance across age and education** ... 19
Table S9: Measurement invariance tests across age (median split at 70.04 years) for the brain-only MTMM-model ... 19
Table S10: Measurement invariance tests across education (median split at 13 years) for the brain-only MTMM-model ... 19
6. **References** ... 20

1. Episodic memory task descriptions

Verbal Learning and Memory test (VLMT) The VLMT is a verbal list learning task that was used to assess recall of words after deliberate learning. Participants are presented a list of 15 words auditory via headphones for 5 times (always in the same order). After each presentation of the word list, participants are instructed to enter all words they can recall into the computer via keyboard. They enter the words one by one followed by enter, which lets the current word disappear so that only one word is visible at the time. After 5 times the word list, an interference list with 15 new words is presented and the participants are again requested to enter all words they can recall. Subsequently, participants are requested to enter all words they can recall from the first list, without the first list being presented again (free recall after distraction). After 30 minutes of performing other tasks, participants are asked to recall as many words as possible from the first list again (delayed recall), followed by a recognition test. In the recognition test, they were presented with all words from the first and the second list plus 20 new words, consisting of 5 of each of the following: 1) synonyms to words from the first and 2) from the second list, and 3) orthographically similar words to words from the first and 2) from the second list. The VLMT took about 30 minutes in total. After one week (same time of the day, seven days later), participants came back for further assessments, and were asked to recall and enter the words from the first list again, without the list being presented again. For the purpose of this study, we used the sum of correctly recalled words across the responses to all 5 learning trials.

Face-Profession task The face profession task was used to assess associative memory recognition performance after incidental encoding. After 2 practice trials with encoding and recall, participants were requested to view 45 face-profession pairs and rate whether the face matches the profession. Next, they were given a distraction task in which they had to count down in intervals of three from 5679 for two minutes. After that, they were asked to watch a series of 54 face-profession pairs and to indicate for each pair whether they had seen the pair

before. After their answer, they were asked to rate their confidence in the answer (not sure, quite sure, very sure). The set of 54 pairs in the recognition phase consisted of 27 old pairs, 9 new pairs, and 18 newly arranged pairs. For the purpose of this study, we used the recognition score (hits minus false alarms).

Scene Encoding. The scene encoding task was used to assess recognition of incidentally encoded scenes. Participants were presented with a series of grey-scale photos of indoor and outdoor scenes (500*300 pixels resolution, mean grey value 127. SD = 75. neutral valence). each presented for 2.5 s. Participants were instructed to decide for each scene whether it was indoor (button press with left index finger) or outdoor (right index finger). After 10 practice trials with each 5 indoor and outdoor scenes, participants were asked to view and judge 88 scenes (44 of each type). Then, they were presented with 44 of the seen items (22 indoor. 22 outdoor) randomly intermixed with 44 new items (22 indoor, 22 outdoor), and asked to indicate how confident they are that they had seen the scene before (on a scale from 1 = sure that new to 5 = sure that old). There was no time restriction on the response. After 2.5 hours, the recognition test was repeated (delayed recognition). In this study, we used the recognition score (hits minus false alarms) from the delayed recognition test.

Object-Location task The object-location task was used to assess recall of deliberately encoded object locations. Sequences of 12 photographs of real-world objects were displayed at different locations in a 6 by 6 locations grid. After the sequence, objects appeared at the side of the screen and participants were asked to move pictures to the location they were first presented at, by means of clicking the picture and the location with the mouse. The task consisted of one practice trial and two test trials. The sum of correct placements across the two test trials is used in this study.

2. Fit indices and parameter estimates of final models

Tables S1-S3 contain the fit indices and parameter estimates, their standard errors of estimation, standardized estimates and their standard errors, z-values and p-values as in lavaan (Rosseel 2012) output. Table S1 contains estimates for the correlation model, Table S2 for the correlation model with covariates, and S3 for the regression model.

Table S1. Fit indices and parameter estimates for the correlation model

Index / parameter	Value
Chi squared	203.268
Degrees of freedom	120
P-value of chi squared	< 0.001
Chi squared of baseline model	3,245.94
Degrees of freedom of baseline model	171
P-value of chi squared of baseline model	< 0.001
CFI	0.973
TLI	0.961
Loglikelihood	-19,868.802
AIC	39,915.603
BIC	40,389.717
Total N	1,521
RMSEA	0.021
RMSEA (CI, lower bound)	0.016
RMSEA (CI, upper bound)	0.026
SRMR	0.05

Latent variable	Loading	Indicator	Estimate	SE	Std. estimate	SE	Z-value	P-value*
PFC	=~	Vmo	1	0	0.3323	0.0584	5.6874	< 0.0001
PFC	=~	MTmo	2.07	0.3834	0.6623	0.0559	11.8543	< 0.0001
PFC	=~	MDmo	-0.6148	0.3219	-0.2038	0.1165	-1.7495	0.0802
PFC	=~	Vdl	1.1218	0.1358	0.3726	0.06	6.2061	< 0.0001
PFC	=~	MTdl	1.1383	0.2795	0.364	0.0832	4.3741	< 0.0001
PFC	=~	MDdl	-2.3444	0.4199	-0.7567	0.0729	-10.3746	< 0.0001
HC	=~	Vhc	1	0	0.6333	0.0375	16.8983	< 0.0001
HC	=~	MThc	1.5598	0.1177	0.9506	0.0143	66.6458	< 0.0001
HC	=~	MDhc	-1.5913	0.1212	-0.9263	0.0148	-62.4141	< 0.0001
PHG	=~	Vphg	1	0	0.4336	0.0527	8.2296	< 0.0001
PHG	=~	MTphg	1.8674	0.2531	0.7707	0.0343	22.4696	< 0.0001
PHG	=~	MDphg	-2.0053	0.2606	-0.8158	0.0376	-21.7207	< 0.0001
PRE	=~	Vpre	1	0	0.4425	0.0523	8.4581	< 0.0001
PRE	=~	MTpre	1.6027	0.2064	0.6824	0.0476	14.3424	< 0.0001

PRE	=~	MDpre	-1.6361	0.2317	-0.7244	0.0541	-13.3986	< 0.0001
V	=~	Vpre	1	0	0.6043	0.0592	10.2018	< 0.0001
V	=~	Vphg	0.657	0.1301	0.3984	0.0607	6.5674	< 0.0001
V	=~	Vhc	0.2986	0.1058	0.1799	0.0601	2.9942	0.0028
V	=~	Vdl	0.8407	0.1498	0.5063	0.0644	7.859	< 0.0001
V	=~	Vmo	0.9922	0.1625	0.5978	0.0598	9.9962	< 0.0001
MT	=~	MTpre	1	0	0.637	0.0443	14.3831	< 0.0001
MT	=~	MTphg	0.4962	0.1002	0.3138	0.0625	5.0176	< 0.0001
MT	=~	MThc	0.3403	0.0733	0.2161	0.0474	4.5594	< 0.0001
MT	=~	MTdl	1.2148	0.1224	0.7716	0.0446	17.3122	< 0.0001
MT	=~	MTmo	0.7263	0.1188	0.4616	0.0703	6.5672	< 0.0001
MD	=~	MDpre	1	0	0.6138	0.0447	13.7323	< 0.0001
MD	=~	MDphg	0.6985	0.0789	0.4035	0.0589	6.8461	< 0.0001
MD	=~	MDhc	0.2055	0.0804	0.1155	0.0494	2.3406	0.0193
MD	=~	MDdl	0.9343	0.1074	0.5552	0.0828	6.706	< 0.0001
MD	=~	MDmo	1.6044	0.1159	0.979	0.0242	40.3839	< 0.0001
EM	=~	VLMT	1	0	0.5239	0.0301	17.3881	< 0.0001
EM	=~	FP	0.8742	0.084	0.4579	0.0305	15.0338	< 0.0001
EM	=~	SE	0.9715	0.0905	0.5089	0.0301	16.8912	< 0.0001
EM	=~	OL	1.0761	0.097	0.5635	0.0303	18.6193	< 0.0001
**Indicator**	**Residual variance**		**Estimate**	**SE**	**Std. estimate**	**SE**	**Z-value**	**P-value***
Vmo	~~	Vmo	2.093	0.2715	0.5322	0.0678	7.8498	< 0.0001
MTmo	~~	MTmo	1.4776	0.2044	0.3483	0.0523	6.6565	< 0.0001
MDmo	~~	MDmo	0	0	0	0	NA	NA
Vdl	~~	Vdl	2.3806	0.264	0.6048	0.0629	9.6223	< 0.0001
MTdl	~~	MTdl	1.1559	0.1923	0.2722	0.0493	5.5171	< 0.0001
MDdl	~~	MDdl	0.4966	0.1706	0.1191	0.0428	2.7852	0.0054
Vhc	~~	Vhc	2.2289	0.1938	0.5665	0.0458	12.3592	< 0.0001
MThc	~~	MThc	0.2112	0.09	0.0497	0.0218	2.2783	0.0227
MDhc	~~	MDhc	0.5991	0.1103	0.1286	0.0257	5.0092	< 0.0001
Vphg	~~	Vphg	2.5366	0.238	0.6533	0.0531	12.3133	< 0.0001
MTphg	~~	MTphg	1.3177	0.1775	0.3075	0.046	6.6874	< 0.0001
MDphg	~~	MDphg	0.757	0.147	0.1716	0.0367	4.681	< 0.0001
Vpre	~~	Vpre	1.7159	0.2655	0.4389	0.0688	6.3756	< 0.0001
MTpre	~~	MTpre	0.5425	0.243	0.1285	0.0601	2.1376	0.0325
MDpre	~~	MDpre	0.3844	0.2004	0.0984	0.0516	1.9063	0.0566
VLMT	~~	VLMT	2.8976	0.145	0.7255	0.0316	22.9837	< 0.0001
FP	~~	FP	3.1578	0.1421	0.7904	0.0279	28.339	< 0.0001
SE	~~	SE	2.9595	0.1439	0.741	0.0307	24.1605	< 0.0001
OL	~~	OL	2.7276	0.149	0.6825	0.0341	20.0075	< 0.0001
**Latent variable**	**Variance**		**Estimate**	**SE**	**Std. estimate**	**SE**	**Z-value**	**P-value***
PFC	~~	PFC	0.4342	0.1604	1	0	NA	NA
HC	~~	HC	1.578	0.2567	1	0	NA	NA

PHG	~~	PHG	0.73	0.1952	1	0	NA	NA
PRE	~~	PRE	0.7656	0.1968	1	0	NA	NA
V	~~	V	1.4277	0.3156	1	0	NA	NA
MD	~~	MD	1.4715	0.2722	1	0	NA	NA
MT	~~	MT	1.7136	0.3128	1	0	NA	NA
EM	~~	EM	1.0961	0.1407	1	0	NA	NA

Latent variable	Covariance	Latent variable	Estimate	SE	Std. estimate	SE	Z-value	P-value*
PFC	~~	HC	0.5263	0.1279	0.6358	0.049	12.9892	< 0.0001
PFC	~~	PHG	0.4038	0.1149	0.7173	0.0497	14.4443	< 0.0001
PFC	~~	PRE	0.177	0.09	0.3069	0.1064	2.8851	0.0039
HC	~~	PHG	0.909	0.1819	0.847	0.0279	30.3332	< 0.0001
HC	~~	PRE	0.289	0.111	0.263	0.0813	3.2364	0.0012
PHG	~~	PRE	0.3487	0.103	0.4665	0.0767	6.0822	< 0.0001
V	~~	MT	0.2081	0.1823	0.133	0.1089	1.2215	0.2219
V	~~	MD	-0.6893	0.1934	-0.4755	0.0788	-6.0358	< 0.0001
MT	~~	MD	-1.108	0.2327	-0.6978	0.0546	-12.7835	< 0.0001
PFC	~~	EM	0.1678	0.0726	0.2432	0.089	2.7316	0.0063
HC	~~	EM	0.4305	0.1141	0.3274	0.0772	4.2402	< 0.0001
PHG	~~	EM	0.2442	0.0862	0.273	0.086	3.1766	0.0015
PRE	~~	EM	0.1504	0.0872	0.1641	0.0898	1.8285	0.0675
V	~~	EM	0.0832	0.1256	0.0665	0.0998	0.6666	0.505
MT	~~	EM	-0.0264	0.1491	-0.0193	0.109	-0.1768	0.8597
MD	~~	EM	-0.1554	0.1079	-0.1224	0.0821	-1.4907	0.136

Indicator	Residual covariance	Indicator	Estimate	SE	Std. estimate	SE	Z-value	P-value*
Vmo	~~	Vdl	1.388	0.2376	0.6218	0.0464	13.4039	< 0.0001
Vhc	~~	Vphg	1.1347	0.1716	0.4772	0.049	9.7418	< 0.0001

Note. *P-values from Wald test*.

Table S2. Fit indices and parameter estimates for the correlation model with age, education, and sex as covariates

**Index / parameter**	**Value**
Chi squared	334.904
Degrees of freedom	159
P-value of chi squared	< 0.001
Chi squared of baseline model	3.646.261
Degrees of freedom of baseline model	231
P-value of chi squared of baseline model	< 0.001
CFI	0.948
TLI	0.925
Loglikelihood	-28,335.874
AIC	56,903.747
BIC	57,521.77
Total N	1,522
RMSEA	0.027
RMSEA (CI, lower bound)	0.023
RMSEA (CI, upper bound)	0.031
SRMR	0.047

**Latent variable**	**Loading**	**Indicator**	**Estimate**	**SE**	**Std. estimate**	**SE**	**Z-value**	**P-value***
PFC	=~	Vmo	1	0	0.2398	0.0655	3.6627	0.0002
PFC	=~	MTmo	2.4117	0.6293	0.565	0.0786	7.1905	< 0.0001
PFC	=~	MDmo	0.2152	0.5702	0.0522	0.1309	0.3989	0.69
PFC	=~	Vdl	1.2146	0.2036	0.2915	0.0684	4.2593	< 0.0001
PFC	=~	MTdl	0.8165	0.4185	0.1913	0.1079	1.7732	0.0762
PFC	=~	MDdl	-2.5262	0.6667	-0.5983	0.0993	-6.024	< 0.0001
HC	=~	Vhc	1	0	0.6096	0.0398	15.3196	< 0.0001
HC	=~	MThc	1.5555	0.1233	0.9215	0.0211	43.6729	< 0.0001
HC	=~	MDhc	-1.608	0.1289	-0.9077	0.0202	-45.0454	< 0.0001
PHG	=~	Vphg	1	0	0.3651	0.0569	6.4215	< 0.0001
PHG	=~	MTphg	2.03	0.3337	0.7155	0.044	16.2599	< 0.0001
PHG	=~	MDphg	-2.155	0.3499	-0.7494	0.0473	-15.841	< 0.0001
PRE	=~	Vpre	1	0	0.3918	0.0514	7.6153	< 0.0001
PRE	=~	MTpre	1.7448	0.25	0.6646	0.0529	12.5531	< 0.0001
PRE	=~	MDpre	-1.7383	0.2808	-0.6871	0.0526	-13.0508	< 0.0001
V	=~	Vpre	1	0	0.5979	0.0583	10.262	< 0.0001
V	=~	Vphg	0.6456	0.1208	0.3881	0.0595	6.5277	< 0.0001
V	=~	Vhc	0.2954	0.1009	0.1775	0.0582	3.0504	0.0023
V	=~	Vdl	0.952	0.1753	0.5677	0.0689	8.2428	< 0.0001
V	=~	Vmo	1.0983	0.1858	0.6545	0.0633	10.3385	< 0.0001
MT	=~	MTpre	1	0	0.6228	0.0443	14.0531	< 0.0001
MT	=~	MTphg	0.5133	0.1069	0.3191	0.0645	4.9486	< 0.0001
MT	=~	MThc	0.3344	0.0754	0.2092	0.0469	4.4631	< 0.0001

MT	=~	MTdl	1.2851	0.1323	0.8014	0.0447	17.9367	< 0.0001
MT	=~	MTmo	0.8151	0.1362	0.5083	0.0773	6.573	< 0.0001
MD	=~	MDpre	1	0	0.6086	0.0427	14.2647	< 0.0001
MD	=~	MDphg	0.7038	0.0854	0.4065	0.0579	7.024	< 0.0001
MD	=~	MDhc	0.202	0.0823	0.1134	0.0491	2.3091	0.0209
MD	=~	MDdl	1.0429	0.123	0.6192	0.0852	7.2679	< 0.0001
MD	=~	MDmo	1.6575	0.1231	1.0077	0.0163	61.7664	< 0.0001
EM	=~	VLMT	1	0	0.5495	0.029	18.9624	< 0.0001
EM	=~	FP	0.8686	0.0797	0.4773	0.0294	16.2153	< 0.0001
EM	=~	SE	0.9005	0.0811	0.4948	0.0292	16.9621	< 0.0001
EM	=~	OL	0.9767	0.0842	0.5365	0.0291	18.4371	< 0.0001
**Indicator**	**Residual variance**		**Estimate**	**SE**	**Std. estimate**	**SE**	**Z-value**	**P-value***
Vmo	~~	Vmo	1.8862	0.2981	0.4755	0.0757	6.2843	< 0.0001
MTmo	~~	MTmo	1.4246	0.203	0.3427	0.0524	6.5442	< 0.0001
MDmo	~~	MDmo	0	0	0	0	NA	NA
Vdl	~~	Vdl	2.1877	0.2848	0.5521	0.0705	7.8266	< 0.0001
MTdl	~~	MTdl	1.1581	0.1968	0.2786	0.0511	5.4503	< 0.0001
MDdl	~~	MDdl	0.5335	0.171	0.1311	0.0438	2.9944	0.0027
Vhc	~~	Vhc	2.2388	0.1933	0.5736	0.0454	12.6277	< 0.0001
MThc	~~	MThc	0.2376	0.0915	0.0575	0.0228	2.522	0.0117
MDhc	~~	MDhc	0.5899	0.1132	0.1296	0.0267	4.8598	< 0.0001
Vphg	~~	Vphg	2.6562	0.2395	0.6814	0.0513	13.2753	< 0.0001
MTphg	~~	MTphg	1.3535	0.1835	0.3236	0.0478	6.7732	< 0.0001
MDphg	~~	MDphg	0.7265	0.1539	0.1691	0.0385	4.3945	< 0.0001
Vpre	~~	Vpre	1.8197	0.2613	0.4618	0.067	6.8894	< 0.0001
MTpre	~~	MTpre	0.5296	0.2653	0.127	0.0656	1.9364	0.0528
MDpre	~~	MDpre	0.4078	0.2216	0.1053	0.0573	1.8388	0.0659
VLMT	~~	VLMT	2.7933	0.1423	0.6981	0.0318	21.9231	< 0.0001
FP	~~	FP	3.0899	0.1398	0.7722	0.0281	27.486	< 0.0001
SE	~~	SE	3.0212	0.1398	0.7551	0.0289	26.1558	< 0.0001
OL	~~	OL	2.8522	0.1418	0.7122	0.0312	22.8145	< 0.0001
**Latent variable**	**Variance**		**Estimate**	**SE**	**Std. estimate**	**SE**	**Z-value**	**P-value***
PFC	~~	PFC	0.1831	0.0983	0.8026	0.0581	13.8233	< 0.0001
HC	~~	HC	1.2159	0.204	0.8384	0.041	20.4462	< 0.0001
PHG	~~	PHG	0.4179	0.1379	0.8042	0.0519	15.4984	< 0.0001
PRE	~~	PRE	0.5695	0.1566	0.9416	0.0336	27.9888	< 0.0001
V	~~	V	1.29	0.2909	0.9157	0.044	20.8158	< 0.0001
MD	~~	MD	1.1645	0.1938	0.8122	0.0523	15.5276	< 0.0001
MT	~~	MT	1.4445	0.2613	0.8933	0.0562	15.8917	< 0.0001
EM	~~	EM	1.0217	0.1237	0.8458	0.0249	33.9061	< 0.0001
**Covariate**	**Variance**		**Estimate**	**SE**	**Std. estimate**	**SE**	**Z-value**	**P-value***
age	~~	age	14.7171	0.5353	1	0	NA	NA
edu	~~	edu	8.3518	0.3226	1	0	NA	NA

sex	~~	sex	0.2499	0.0091	1	0	NA	NA
**Latent variable**	**Covariance**	**Latent variable**	**Estimate**	**SE**	**Std. estimate**	**SE**	**Z-value**	**P-value***
PFC	~~	HC	0.2464	0.0819	0.5221	0.0614	8.5035	< 0.0001
PFC	~~	PHG	0.1569	0.0635	0.5671	0.0744	7.6242	< 0.0001
PFC	~~	PRE	0.0028	0.0406	0.0088	0.1246	0.0707	0.9436
HC	~~	PHG	0.5678	0.133	0.7965	0.0386	20.6407	< 0.0001
HC	~~	PRE	0.0461	0.0719	0.0554	0.085	0.6516	0.5147
PHG	~~	PRE	0.1372	0.0549	0.2811	0.0839	3.3503	0.0008
V	~~	MT	0.1177	0.1432	0.0862	0.1022	0.8439	0.3987
V	~~	MD	-0.5668	0.1497	-0.4624	0.0746	-6.1989	< 0.0001
MT	~~	MD	-0.8628	0.163	-0.6652	0.0541	-12.2991	< 0.0001
PFC	~~	EM	0.0232	0.0436	0.0536	0.0997	0.5374	0.591
HC	~~	EM	0.2111	0.0988	0.1894	0.0861	2.1999	0.0278
PHG	~~	EM	0.0839	0.064	0.1283	0.0959	1.3375	0.1811
PRE	~~	EM	0.0529	0.0711	0.0694	0.0926	0.7495	0.4536
**Indicator**	**Covariance**	**Indicator**	**Estimate**	**SE**	**Std. estimate**	**SE**	**Z-value**	**P-value***
Vmo	~~	Vdl	1.1867	0.2644	0.5842	0.0594	9.8385	< 0.0001
Vhc	~~	Vphg	1.1891	0.1719	0.4876	0.0474	10.288	< 0.0001
**Latent variable**	**Regression path**	**Covariate**	**Estimate**	**SE**	**Std. estimate**	**SE**	**Z-value**	**P-value***
EM	~	age	-0.0632	0.0098	-0.2206	0.032	-6.8935	< 0.0001
EM	~	sex	0.3162	0.0742	0.1438	0.032	4.4978	< 0.0001
EM	~	edu	0.1108	0.0145	0.2913	0.0333	8.7464	< 0.0001
PFC	~	age	-0.0351	0.0143	-0.2816	0.0693	-4.0616	< 0.0001
PFC	~	sex	0.3236	0.1187	0.3386	0.0631	5.3704	< 0.0001
PFC	~	edu	0.0097	0.011	0.0589	0.0651	0.9052	0.3654
HC	~	age	-0.0887	0.0189	-0.2825	0.0524	-5.3912	< 0.0001
HC	~	sex	0.6874	0.1475	0.2854	0.0539	5.2984	< 0.0001
HC	~	edu	0.0081	0.0236	0.0194	0.0565	0.3429	0.7317
PHG	~	age	-0.0512	0.0151	-0.2727	0.0617	-4.4232	< 0.0001
PHG	~	sex	0.5004	0.1173	0.347	0.059	5.8794	< 0.0001
PHG	~	edu	-0.0078	0.0153	-0.0311	0.0612	-0.5082	0.6113
PRE	~	age	-0.013	0.0156	-0.0641	0.0746	-0.8594	0.3901
PRE	~	sex	0.3097	0.1288	0.1991	0.0733	2.7159	0.0066
PRE	~	edu	-0.0326	0.0178	-0.1211	0.0647	-1.8734	0.061
V	~	age	-0.0339	0.0235	-0.1097	0.0732	-1.4978	0.1342
V	~	sex	0.6368	0.1875	0.2682	0.0746	3.596	0.0003
V	~	edu	0.0074	0.03	0.018	0.0727	0.2481	0.804
MT	~	age	-0.0896	0.0282	-0.2702	0.0769	-3.5137	0.0004
MT	~	sex	0.4659	0.2188	0.1832	0.0819	2.237	0.0253
MT	~	edu	0.0048	0.0324	0.0109	0.0736	0.1475	0.8827
MD	~	age	0.1226	0.0226	0.3928	0.0543	7.2397	< 0.0001
MD	~	sex	-0.4383	0.1717	-0.183	0.0654	-2.7979	0.0051
MD	~	edu	0.0029	0.0236	0.0069	0.057	0.1207	0.9039

Note. P-values from Wald test.

Table S3. Fit indices and parameter estimates for the regression model

Index / parameter	Value
Chi squared	206.765
Degrees of freedom	123
P-value of chi squared	< 0.001
Chi squared of baseline model	3,245.94
Degrees of freedom of baseline model	171
P-value of chi squared of baseline model	< 0.001
CFI	0.973
TLI	0.962
Loglikelihood	-19,870.55
AIC	39,913.101
BIC	40,371.234
Total N	0.021
RMSEA	0.016
RMSEA (CI, lower bound)	0.026
RMSEA (CI, upper bound)	0.053

Latent variable	Loading	Indicator	Estimate	SE	Std. estimate	SE	Z-value	P-value*
PFC	=~	Vmo	1	0	0.3369	0.0577	5.8343	< 0.0001
PFC	=~	MTmo	2.0684	0.3775	0.67	0.054	12.4185	< 0.0001
PFC	=~	MDmo	-0.6783	0.3114	-0.2276	0.1137	-2.0022	0.0453
PFC	=~	Vdl	1.1227	0.1339	0.378	0.0592	6.389	< 0.0001
PFC	=~	MTdl	1.1691	0.2765	0.3781	0.0808	4.6779	< 0.0001
PFC	=~	MDdl	-2.3575	0.4147	-0.771	0.0698	-11.0431	< 0.0001
HC	=~	Vhc	1	0	0.6357	0.0371	17.1327	< 0.0001
HC	=~	MThc	1.556	0.1168	0.9515	0.0138	68.9803	< 0.0001
HC	=~	MDhc	-1.5891	0.1204	-0.9281	0.0145	-63.9455	< 0.0001
PHG	=~	Vphg	1	0	0.4364	0.0523	8.3431	< 0.0001
PHG	=~	MTphg	1.8625	0.2506	0.7736	0.0336	22.9921	< 0.0001
PHG	=~	MDphg	-2.0076	0.2588	-0.8212	0.0364	-22.5797	< 0.0001
PRE	=~	Vpre	1	0	0.445	0.0523	8.5118	< 0.0001
PRE	=~	MTpre	1.5962	0.2037	0.6828	0.047	14.516	< 0.0001
PRE	=~	MDpre	-1.646	0.2303	-0.7331	0.053	-13.8428	< 0.0001
V	=~	Vpre	1	0	0.603	0.0602	10.0214	< 0.0001
V	=~	Vphg	0.6521	0.1311	0.3942	0.0606	6.5087	< 0.0001
V	=~	Vhc	0.2901	0.1053	0.1742	0.0595	2.9291	0.0034
V	=~	Vdl	0.8311	0.1503	0.4991	0.0643	7.7611	< 0.0001
V	=~	Vmo	0.9884	0.1644	0.5939	0.0599	9.9219	< 0.0001
MT	=~	MTpre	1	0	0.6381	0.0442	14.4394	< 0.0001

MT	=~	MTphg	0.4816	0.0989	0.305	0.0615	4.9633	< 0.0001
MT	=~	MThc	0.3283	0.0712	0.2088	0.0456	4.5779	< 0.0001
MT	=~	MTdl	1.2023	0.1219	0.7636	0.0458	16.6709	< 0.0001
MT	=~	MTmo	0.7061	0.1176	0.4492	0.0694	6.4684	< 0.0001
MD	=~	MDpre	1	0	0.6067	0.0454	13.3674	< 0.0001
MD	=~	MDphg	0.6883	0.0788	0.3921	0.0577	6.7965	< 0.0001
MD	=~	MDhc	0.1874	0.0786	0.104	0.0472	2.2025	0.0276
MD	=~	MDdl	0.9143	0.1081	0.5363	0.0824	6.5103	< 0.0001
MD	=~	MDmo	1.6182	0.1171	0.9738	0.0266	36.6593	< 0.0001
EM	=~	VLMT	1	0	0.5245	0.0302	17.373	< 0.0001
EM	=~	FP	0.8709	0.0838	0.4567	0.0305	14.9907	< 0.0001
EM	=~	SE	0.969	0.0904	0.5083	0.0302	16.8429	< 0.0001
EM	=~	OL	1.0787	0.0975	0.5656	0.0303	18.6503	< 0.0001
**Indicator**	**Residual variance**		**Estimate**	**SE**	**Std. estimate**	**SE**	**Z-value**	**P-value***
Vmo	~~	Vmo	2.0977	0.2712	0.5338	0.0676	7.8977	< 0.0001
MTmo	~~	MTmo	1.4845	0.2045	0.3493	0.0523	6.6851	< 0.0001
MDmo	~~	MDmo	0	0	0	0	NA	NA
Vdl	~~	Vdl	2.392	0.2631	0.608	0.0624	9.7452	< 0.0001
MTdl	~~	MTdl	1.1682	0.192	0.274	0.0492	5.5727	< 0.0001
MDdl	~~	MDdl	0.4917	0.1711	0.1179	0.0429	2.7518	0.0059
Vhc	~~	Vhc	2.2257	0.1932	0.5655	0.0457	12.3665	< 0.0001
MThc	~~	MThc	0.2168	0.0899	0.051	0.0218	2.337	0.0194
MDhc	~~	MDhc	0.5958	0.1102	0.1278	0.0256	4.9894	< 0.0001
Vphg	~~	Vphg	2.5399	0.2377	0.6541	0.053	12.351	< 0.0001
MTphg	~~	MTphg	1.3228	0.1778	0.3086	0.046	6.7059	< 0.0001
MDphg	~~	MDphg	0.7599	0.1471	0.1719	0.0366	4.6994	< 0.0001
Vpre	~~	Vpre	1.7107	0.2676	0.4384	0.0694	6.3151	< 0.0001
MTpre	~~	MTpre	0.535	0.2369	0.1267	0.0586	2.1615	0.0307
MDpre	~~	MDpre	0.368	0.1959	0.0945	0.0506	1.8654	0.0621
VLMT	~~	VLMT	2.8961	0.1452	0.7249	0.0317	22.8863	< 0.0001
FP	~~	FP	3.1628	0.1421	0.7914	0.0278	28.4355	< 0.0001
SE	~~	SE	2.9634	0.144	0.7417	0.0307	24.1781	< 0.0001
OL	~~	OL	2.7195	0.1494	0.6801	0.0343	19.829	< 0.0001
**Latent variable**	**Variance**		**Estimate**	**SE**	**Std. estimate**	**SE**	**Z-value**	**P-value***
PFC	~~	PFC	0.4459	0.161	1	0	NA	NA
HC	~~	HC	1.5904	0.2567	1	0	NA	NA
PHG	~~	PHG	0.7395	0.1956	1	0	NA	NA
PRE	~~	PRE	0.7729	0.1978	1	0	NA	NA
V	~~	V	1.4188	0.3175	1	0	NA	NA
MD	~~	MD	1.4345	0.2682	1	0	NA	NA
MT	~~	MT	1.7197	0.3111	1	0	NA	NA
EM	~~	EM	0.9614	0.1397	0.8746	0.0561	15.5882	< 0.0001
**Latent variable**	**Covariance**	**Latent variable**	**Estimate**	**SE**	**Std. estimate**	**SE**	**Z-value**	**P-value***

PFC	~~	HC	0.5368	0.1272	0.6374	0.0484	13.1746	< 0.0001
PFC	~~	PHG	0.4142	0.1148	0.7214	0.0482	14.9594	< 0.0001
PFC	~~	PRE	0.1901	0.0907	0.3238	0.1026	3.1552	0.0016
HC	~~	PHG	0.92	0.1819	0.8483	0.0276	30.7172	< 0.0001
HC	~~	PRE	0.3032	0.1103	0.2735	0.079	3.4622	0.0005
PHG	~~	PRE	0.3601	0.1035	0.4763	0.0745	6.3927	< 0.0001
V	~~	MT	0.1824	0.1813	0.1168	0.1094	1.0671	0.2859
V	~~	MD	-0.6656	0.1902	-0.4665	0.079	-5.9025	< 0.0001
MT	~~	MD	-1.0858	0.2285	-0.6913	0.055	-12.5634	< 0.0001
Vmo	~~	Vdl	1.3973	0.2369	0.6238	0.0459	13.578	< 0.0001
Vhc	~~	Vphg	1.1358	0.1713	0.4777	0.0489	9.7656	< 0.0001
**Latent variable**	**Covariance**	**Latent variable**	**Estimate**	**SE**	**Std. estimate**	**SE**	**Z-value**	**P-value***
EM	~	PFC	0.1315	0.2296	0.0838	0.1459	0.574	0.566
EM	~	HC	0.3158	0.1779	0.3799	0.2148	1.7687	0.0769
EM	~	PHG	-0.1856	0.3496	-0.1522	0.288	-0.5286	0.5971
EM	~	PRE	0.1376	0.135	0.1154	0.1131	1.0203	0.3076

Note. *P-values from Wald test*.

3. Sex differences in variables

For sex differences in all variables of interest, see Table S1. Women outperformed men in verbal memory (VLMT), which could be expected (for a review, see Asperholm et al. 2019). but showed no advantage in the other memory tests. In many of the grey matter integrity variables, women showed values indicating higher integrity (larger prefrontal volumes, higher MT values, lower MD values). The literature on regional grey matter structural differences between sexes is so far mixed. For instance, a meta-analysis suggests the extent of differences in volume and density to depend on the brain region (Ruigrok et al., 2014). It is also not trivial to disentangle whether different ways of accounting for ICV might account for differences between studies in regional sex differences (Voevodskaya et al., 2014; Pintzka et al., 2015).

Table S4: Sex differences in all variables of interest

Variable	Mean male	SD male	Mean female	SD female	P-value
VLMT	8.2479	2.61713	8.7371	2.72146	0.0001
FP	0.2597	0.20769	0.273	0.21147	0.21844
SE	0.2724	0.14168	0.2825	0.13775	0.16199
OL	13.0787	4.17183	13.4245	3.8318	0.0946
Vhc	0.5138	0.04725	0.5339	0.041	0.00005
Vphg	0.4287	0.03235	0.4347	0.03106	0.08857
Vpre	0.3915	0.02953	0.4044	0.03032	0.00015
Vmofc	0.4076	0.03117	0.4278	0.02566	< 0.00001
Vdlpfc	0.4062	0.03012	0.4273	0.02401	< 0.00001
MThc	322.5417	34.6065	347.6178	22.48292	< 0.00001
MTphg	360.006	17.41447	369.0587	11.78399	0.00001
MTpre	331.8518	19.59492	342.1202	17.07309	0.0001
MTmo	350.3291	17.21289	359.4151	15.66446	0.00013
MTdl	297.6277	17.75573	305.0321	27.22796	0.03214
MDhc	0.0014	0.00015	0.0013	0.00011	< 0.00001
MDphg	0.0014	0.00012	0.0013	8.00E-05	< 0.00001
MDpre	0.0012	0.00011	0.0012	0.00011	< 0.00001
MDmo	0.0012	9.00E-05	0.0012	0.00011	0.00356
MDdl	0.0012	8.00E-05	0.0011	8.00E-05	< 0.00001
Age	70.5278	4.10643	70.667	3.56062	0.48231
Education	14.6049	2.87097	13.7467	2.85068	< 0.00001

Note. P-values from Welch T-test for unequal variances.

4. Sex differences in association structures

Figure S1: First-order correlations among all variables by sex. Left: male participants, right: female participants.

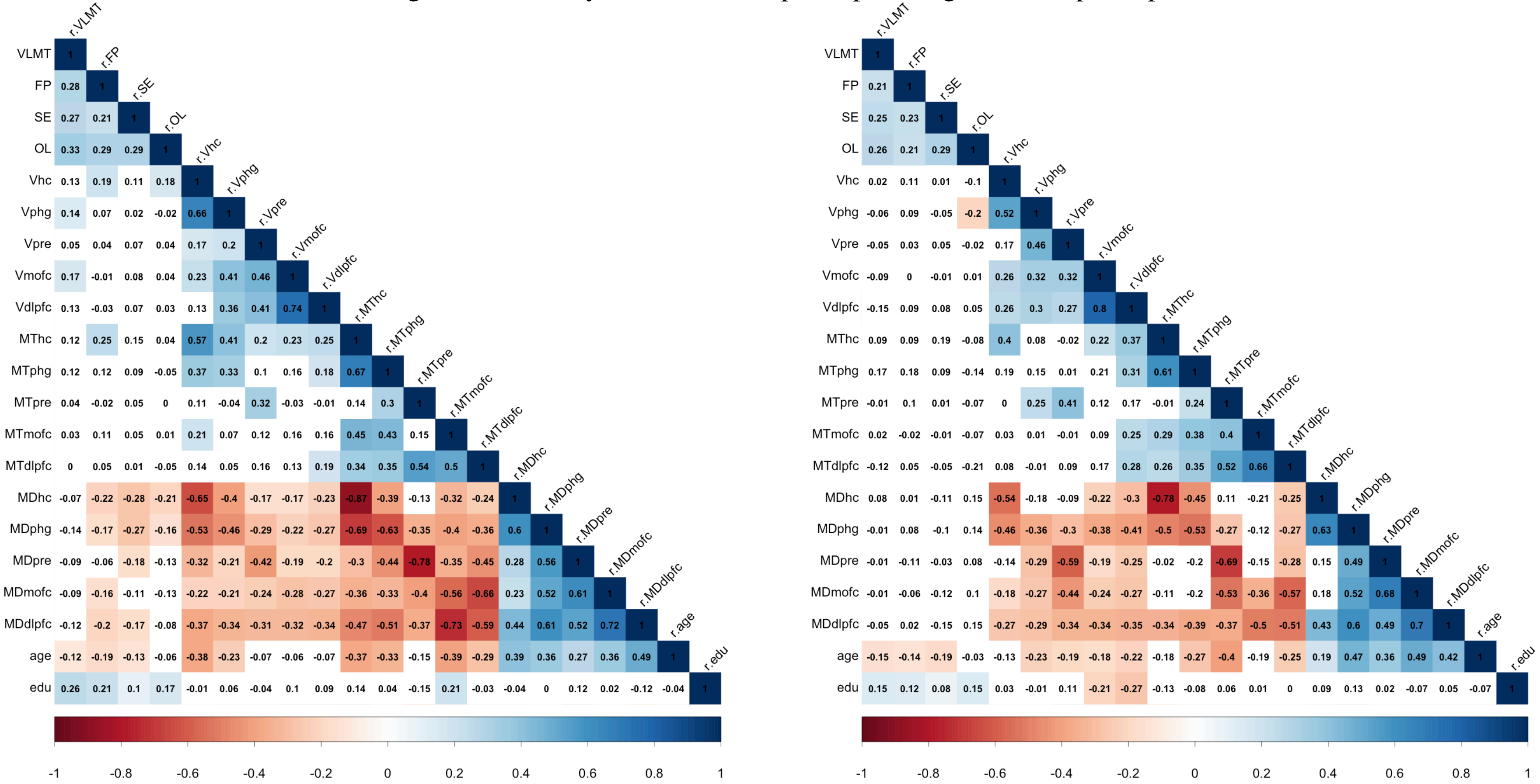

Note. *Color coding according to strength and direction of correlation (blue = positive. red = negative). White background: correlation non-significant (p > .05). VLMT = verbal learning and memory test; FP = face-profession task; SE = scene encoding; OL = object location task; V = VBM (volume-based morphometry); MT = Magnetization tranfer ratio; MD = mean diffusivity; hc = hippocampus; phg = parahippocampal gyrus; pre = precuneus; mofc = medio-orbitofrontal cortex; dlpfc = dorsolateral prefrontal cortex; edu = years of education.*

We tested the brain-only grey matter integrity MTMM for measurement invariance (Meredith 1993. Meredith and Teresi 2006) across sexes in the following way. First, we specified a two-group model in which we allowed all parameters to differ across groups (configural invariance). When we specified exactly the same model as for the total sample, a two-group model did not converge. A pruned version of the model with the non-significant loading of MD in medio-orbitofronal cortex on the PFC factor and MD fixed to zero fit the total sample data comparably well (Chi square difference (df = 2) = 3.56. p = .17). This model was easier to fit to the two groups. For fit indices of the two group model with configural invariance, that is, all parameters allowed to differ, see Table S4.

After fitting the configurally invariant model, we specified a model with all loadings restricted to be estimated at the same value for women and men (metric invariance). We compared the two models by their Chi square-values, checked the Chi square-difference and whether it is significant. Because the Chi square-difference depends on sample size and might be overly sensitive in large samples, we also checked the difference in CFI, RMSEA and SRMR for a relevant difference following rules-of-thumb based on a simulations study (Chen 2007) saying "when sample size is adequate (total N > 300) and sample sizes are equal across groups […]. a CFI difference of -.01, supplemented by a change of >= .015 in RMSEA or a change of >= .03 in SRMR would indicate noninvariance" (Chen 2007). We then fit a model with loadings and residual variances of the indicator variables to be the same across sexes and compared it to the metric invariance model.

The Chi square difference test and the RMSEA suggested a relevant worsening of fit when assuming invariant residuals, as well as compared to metric invariance, but the changes in RMSEA and SRMR did not reach the cutoffs, so that we continued assuming invariance of residuals as well (Table S5).

Table S5: Measurement invariance tests for the brain-only MTMM-model

Invariance level	Df	AIC	BIC	Chi2	Chi2diff	Pr(>Chi2)	CFI	RMSEA	SRMR
same model (configural)	132	14331	14856	221			0.962	0.0638	0.0691
same loadings (metric)	153	14321	14765	252	31.4	0.0680	0.958	0.0626	0.0805
same loadings and residuals	168	14334	14722	295	43.5	0.0001	0.945	0.0678	0.1030

Note. Df: degrees of freedom; AIC: Akaike information criterion; CFI: Comparitive fit index; RMSEA: root mean squared error of approximation; SRMR: standardized root mean square residual

For episodic memory measurement invariance, see Table S6. Here, the above cutoffs suggested invariance of loadings and of residuals.

Table S6: Measurement invariance tests for the episodic memory measurement model

Invariance level	Df	AIC	BIC	Chisq	Chisq diff	Df diff	Pr(>Chi2)	CFI	RMSEA	SRMR
same model (configural)	4	24822	24950	1.0456				1.0000	0.0000	0.0052
same loadings (metric)	7	24819	24931	3.7634	2.7178	3	0.4372	1.0000	0.0000	0.0139
same loadings and residuals	11	24819	24910	12.2636	8.5002	4	0.0749	0.9974	0.0123	0.0276

Note. Df: degrees of freedom; AIC: Akaike information criterion; CFI: Comparitive fit index; RMSEA: root mean squared error of approximation; SRMR: standardized root mean square residual

We fit a multigroup model that includes both the grey matter model and episodic memory. with loadings and residuals fixed to be equal across sexes in the brain model. From this model we then obtained the estimated correlations between episodic memory and each of the grey matter ROIs. Please see Table S7 for the results. The results suggest that there are significant sex differences in the association between hippocampus and episodic memory as well as between parahippocampal gyrus and episodic memory. The associations found in the total sample are restricted to men. When adding age and education into the model (Table S8), the

pattern stays the same, only that these associations are slightly attenuated within the male group.

Table S7: Associations between episodic memory and each of the ROI-wise integrity factors by sex. from a multigroup model

Association of episodic memory with	Men covariance (SE)	Men correlation / std. cov	Women covariance (SE)	Women correlation / std. cov	Chi2 difference when setting covariance equal (df =1)	P-Value from Chi2 difference
PFC	.20 (.10)	.24	-0.004(.09)	-.008	2.4	.12
HC	.58 (.10)	.39	-.042 (.13)	-.02	8.97	.0027
PHG	.27 (.10)	.32	-0.04 (.08)	-.09	6.54	.011
PRE	.08 (.14)	.07	0.10 (.17)	.09	0.00516	.94

Note. PFC: prefrontal cortex; HC: hippocampus; PHG: parahippocampal gyrus; PRE: precuneus

Table S8: Associations between episodic memory and each of the ROI-wise integrity factors by sex. from a multigroup model with age and education as covariates

Association of episodic memory with	Men covariance (SE)	Men correlation / std. cov	Women covariance (SE)	Women correlation / std. cov	Chi2 difference when setting covariance equal (df =1)	P-Value from Chi2 difference
PFC	.02 (.08)	.03	-0.03 (.09)	-.05	0.154	.70
HC	.37 (.14)	.29	-0.04 (.13)	-.05	4.76	.029
PHG	.18 (.09)	.24	-0.06 (.07)	-.16	4.5	.034
PRE	.09 (.13)	.08	0.03 (.17)	.03	0.067	.8

Note. PFC: prefrontal cortex; HC: hippocampus; PHG: parahippocampal gyrus; PRE: precuneus

As this is the result of an ad hoc exploratory analysis, we can only speculate post-hoc about the reasons for the associations to be restricted to men. In spite of this finding, we would not yet refrain from hypothesizing that hippocampal grey matter structure is related to episodic memory performance in later life also in women. We do not believe that fundamentally different processes are at work when it comes to brain integrity and cognitive aging in men and women; therefore, we did not expect these differences and did not consider them when conceptualizing this study. Rather, a larger share of the men than of the women might already have proceeded further on their brain aging trajectory, are beginning to suffer cognitive consequences. To speculate, in our male population, metabolic risk might be more prevalent,

with detrimental effects on both grey matter integrity (Raz et al. 2005; Raz and Rodrigue 2006) and episodic memory (Yates et al. 2012). This might also be the cause of men showing lower mean brain integrity estimates and lower mean episodic memory performance than women in the present study (Tables 3 and S2). Future studies including longitudinal follow-ups and measures of metabolic risk may shed light on these questions.

In response to a reviewer's comment on the sex differences in associations, we formally tested equality of latent factor variances across men and women. Assuming equal variance of the episodic memory factor across groups does not result in a worse fitting model (Chi-square difference (df =1): 2.32. p = .13). Assuming equal variance of the hippocampus latent factor does indeed result in a worse fitting model (Chi-square difference (df =1): 21.11. p = . 0.000004). The same is true for PHG (Chi-square difference (df =1): 18.68. p = . 0.00015) and for PFC (Chi-square difference (df =1): 12.75. p = . 0.00035). but not for precuneus (Chi-square difference (df =1): 0.21. p = . 0.65). That is, men show significantly greater variance in the latent factors for hippocampus, parahippocampal gyrus, and prefrontal cortex, but not episodic memory and precuneus. This fits well with our speculation that a larger share of men than women might have already undergone detrimental age-related changes in brain structure with consequences for memory performance. (Acknowledging that, with the cross-sectional data at hand, we cannot distinguish older individuals that have had relatively low grey matter integrity throughout life from those who have already suffered from age-related decline. This questions thus calls for longitudinal data.)

5. Measurement invariance across age and education

Strictly, before we compare older and younger (or more or less educated) participants' parameter with each other, we would also here have to test measurement invariance, as a reviewer pointed out. As a very coarse approximation, and because sample size in the MR subsample limits much finer parcellations of the data, we did a median split in age and education and tested each for measurement invariance. For age, the median was 70.04 years. The two age groups differed in their residual variances according to the chi square difference test. but the other fit indices indicated not much worsening fit by assuming the same residuals (Table S9). For education, median was 13 years. Those with 13 years and more did not differ in loadings nor residuals from those with less than 13 years of education (Table S10).

Table S9: Measurement invariance tests across age (median split at 70.04 years) for the brain-only MTMM-model

Invariance level	Df	AIC	BIC	Chi2	Chi2diff	Pr(>Chi2)	CFI	RMSEA	SRMR
same model (configural)	130	14430	14962	227			0.960	0.0672	0.0688
same loadings (metric)	153	14412	14857	255	27.8	0.2255	0.958	0.0634	0.0772
same loadings and residuals	168	14414	14806	285	30.1	0.0073	0.951	0.0653	0.0798

Note. Df: degrees of freedom; AIC: Akaike information criterion; CFI: Comparitive fit index; RMSEA: root mean squared error of approximation; SRMR: standardized root mean square residual

Table S10: Measurement invariance tests across education (median split at 13 years) for the brain-only MTMM-model

Invariance level	Df	AIC	BIC	Chi2	Chi2diff	Pr(>Chi2)	CFI	RMSEA	SRMR
same model (configural)	130	12955	13471	204			0.966	0.062	0.054
same loadings (metric)	153	12932	13363	226	22.7	0.479	0.966	0.057	0.063
same loadings and residuals	168	12918	13298	241	14.2	0.438	0.966	0.055	0.065

Note. Df: degrees of freedom; AIC: Akaike information criterion; CFI: Comparitive fit index; RMSEA: root mean squared error of approximation; SRMR: standardized root mean square residual

6. References

Asperholm M. Högman N. Rafi J. Herlitz A. 2019. What did you do yesterday? A meta-analysis of sex differences in episodic memory. Psychol. Bull. 145:785–821.

Chen FF. 2007. Sensitivity of goodness of fit indexes to lack of measurement invariance. Struct. Equ. Model. 14:464–504.

Meredith W. 1993. Measurement invariance. factor analysis and factorial invariance. Psychometrika 58:525–543.

Meredith W. Teresi JA. 2006. An essay on measurement and factorial invariance. Med. Care 44:S69–S77.

Pintzka CWS. Hansen TI. Evensmoen HR. Håberg AK. 2015. Marked effects of intracranial volume correction methods on sex differences in neuroanatomical structures: a HUNT MRI study. Front. Neurosci. 9:1–17.

Raz N. Lindenberger U. Rodrigue KM. Kennedy KM. Head D. Williamson A. Dahle C. Gerstorf D. Acker JD. 2005. Regional brain changes in aging healthy adults: General trends. individual differences and modifiers. Cereb. Cortex 15:1676–1689.

Raz N. Rodrigue KM. 2006. Differential aging of the brain: Patterns. cognitive correlates and modifiers. Neurosci. Biobehav. Rev. 30:730–748.

Rosseel Y. 2012. lavaan: An R Package for Structural Equation Modeling. Journal of Statistical Software 48:2.

Ruigrok ANV. Salimi-Khorshidi G. Lai M-C. Baron-Cohen S. Lombardo MV. Tait RJ. Suckling J. 2014. A meta-analysis of sex differences in human brain structure. Neurosci. Biobehav. Rev. 39:34–50.

Voevodskaya O. Simmons A. Nordenskjold R. Kullberg J. Ahlstrom H. Lind L. Wahlund L-O. Larsson E-M. Westman E. 2014. The effects of intracranial volume adjustment

approaches on multiple regional MRI volumes in healthy aging and Alzheimer's disease. Front. Aging Neurosci. 6:264.

Yates KF. Sweat V. Yau PL. Turchiano MM. Convit A. 2012. Impact of metabolic syndrome on cognition and brain: A selected review of the literature. Arteriosclerosis. Thrombosis. and Vascular Biology 32:2060–2067.
